# Ubiquitin-related lncRNAs: The new tool for prognosis prediction in prostate cancer

**DOI:** 10.3389/fonc.2022.948113

**Published:** 2022-09-13

**Authors:** Xiang Liu, Wangli Mei, Liang Jin, Xianchao Sun, Zhen Zhou, Shiyong Xin, Liqun Huang, Guosheng Yang, Jinyou Wang, Lin Ye

**Affiliations:** ^1^ Department of Urology, Putuo People’s Hospital, School of Medicine, Tongji University, Shanghai, China; ^2^ Department of Urology, Shanghai East Hospital, School of Medicine, Tongji University, Shanghai, China; ^3^ Department of Urology, Shanghai Tenth People's Hospital, School of Medicine, Tongji University, Shanghai, China; ^4^ Department of Urology, The Second Affiliated Hospital of Anhui Medical University, Hefei, Anhui, China

**Keywords:** prostate cancer, ubiquitin, lncRNA, prognosis, progression-free survival

## Abstract

**Objective:**

To establish a ubiquitin-related long noncoding ribonucleic acids (lncRNAs) prognosis prediction model for prostate cancer (Pca).

**Methods:**

Data were acquired through The Cancer Genome Atlas (TCGA) database. Ubiquitin-related differentially expressed genes (DEGs) and lncRNAs in Pca were filtered out. UBE2S was selected as the representative gene and validated *in vitro*. Progression-free survival (PFS) predictive signature was established with ubiquitin-related lncRNAs screened by Cox regression analyses and internally validated. A nomogram was constructed to assess the prognosis of Pca patients. Gene enrichment analysis was performed to explore functional differences based on risk stratification. Between different risk groups, immune status and drug sensitivity were contrasted.

**Results:**

A total of 254 ubiquitin-related genes were screened. UBE2S was shown to promote the proliferation of Pca cells *in vitro*. The predictive signature was established based on six ubiquitin-related lncRNAs and validated. The prognosis of Pca patients was worse with an increasing risk score. The area under the curve (AUC) of the signature was higher than that of clinicopathological variables (0.806 vs 0.504–0.701). The AUC was 0.811 for 1-year PFS, 0.807 for 3-year PFS, and 0.790 for 5-year PFS. The calibration curves of risk score-based nomogram demonstrated high consistency. By contrasting the expression of immune function, cells, and checkpoints, we found that the signature was closely related to immunity. The high-risk patients were more sensitive to gemcitabine, cisplatin, bortezomib, etc. and resistant to bicalutamide.

**Conclusion:**

The ubiquitin-related lncRNAs can effectively predict the prognosis of Pca and may provide new treatment options for Pca.

## Introduction

Prostate cancer (Pca) is the second leading cancer in men worldwide. It accounts for 14.1% of new cases and 6.8% of total mortality in male cancer around the globe in 2020 (1). Even after undergoing radical prostatectomy, about 40% patients with Pca ultimately progress to biochemical recurrence (2). Many studies have been conducted to solve this problem. It is commonly believed that Pca is unique in its dependence on androgen for progression, and androgen deprivation is a therapeutic strategy widely used in clinical practice (3). However, most patients may progress through the androgen-sensitive stage to castration-resistant prostate cancer (CRPC) with poorer prognosis in 2–3 years (4). It will reveal the molecular mechanism and give patients individualized precision therapy strategy by identifying specific genes with prognostic and therapeutic potential for Pca. Ubiquitin is a 76-amino acid protein and involved in multiple post-translational modifications (5). Ubiquitin regulates many important cellular processes by E1, E2, and E3 enzymes, which participate in activation, conjugation, and ligation, respectively (6). The ubiquitin-related gene family is believed to participate in the pathogenesis of various human tumors. In immune regulation, ubiquitin ligase Cbl-b has been identified as a key regulation factor in T-cell activation and tolerance (7). In the field of autophagy, TRAF6 assembles ubiquitin chains on ULK1 promoting its stabilization, self-association, and autophagy, and subsequently regulates the mTOR pathway (8). In cell-cycle regulation, deregulation of ubiquitin ligases may degrade cyclins and cyclin-dependent kinase inhibitor proteins thus promote the proliferation of cancer cells (9). Furthermore, the ubiquitin family is involved in classical tumor-related signal pathways such as the MAPK pathway (10, 11) and the PI3K-AKT-mTOR pathway (12, 13). Therefore, we believe that the ubiquitin family takes a key position in the pathogenesis of Pca.

Long noncoding ribonucleic acids (lncRNAs) are non-coding RNA molecules with a length over 200 bp (14). LncRNAs with polyadenylation resemble the messenger RNA (mRNA) consensus sequence (15), which may execute cytoplasmic functions such as microRNA (miRNA) sponging, interact with signaling proteins, and regulate the translation of mRNAs (16). The huge number and large diversity of functions of different lncRNAs present many opportunities for tumor regulation (17). For example, lncRNA-p21 was initially identified as a tumor suppressor induced by p53 and was proved to promote the transcription of gene CDKN1A (18, 19). Moreover, lncRNA BCRT1 serves as a miRNA-1303 regulator, participating in the breast cancer regulatory pathway (20).

However, the role of ubiquitin-related lncRNAs in the prognosis of Pca was unrevealed until now. To fill this gap, we created the ubiquitin-related lncRNAs signature for Pca patients with the aim to predict the prognosis and discover novel therapeutic targets.

## Materials and methods

### Patients and datasets

We downloaded standardized RNA-seq data and the corresponding clinical data from The Cancer Genome Atlas (TCGA) database (https://portal.gdc.cancer.gov/). LncRNA expression values for 554 Pca patients and progression-free survival (PFS) data for 501 Pca patients were obtained from the TCGA and cBioPortal databases (https://www.cbioportal.org/), respectively.

### Screening and functional enrichment analysis of target genes

Genes involved in pathways related to the ubiquitin (21) were downloaded from Genecards (https://www.genecards.org/). A total of 545 genes were adopted with an inclusion criterion of relevance score > 7. The false discovery rate< 0.05 and the log_2_ fold change > 0.95 were used as the screening criteria to screen the differentially expressed genes (DEGs) between Pca and normal tissues. A total of 254 genes were filtered as ubiquitin-related DEGs after the intersection of ubiquitin-related genes and DEGs in Pca. Functional enrichment analysis was calculated with R software version 4.1.3 in the data of Kyoto Encyclopedia of Genes and Genomes (KEGG) and Gene Ontology (GO).

### Biological validation of UBE2S

After pan-cancer analysis of ubiquitin-related DEG expression in the TCGA database, UEB2S was chosen as the representative gene for identification. We aimed to investigate whether different expression of UBE2S influences the biological behavior of Pca cells *in vitro*. We used human prostate cancer cells LNCaP (Cell Bank of Shanghai Academy of Chinese Sciences, Shanghai, China) transfected with UBE2S siRNA and control siRNA (GenePharma, Shanghai, China) as observation objects. For the transient transfection, siRNA was mixed with Lipofectamine 3000 (Invitrogen, Shanghai, China) in OptiMEM to form complexes. The efficacy of siRNA knockdown was tested by real-time polymerase chain reaction (PCR). A CCK-8 kit (Tsingke, Shanghai, China) was used to measure the proliferation of LNCaP cells. Scratch-wound-healing assay was employed to investigate the migration. Validation of UBE2S protein expression was done by immunohistochemistry with a routine SABC technique in pathological sections of Pca patients (GloriousMed, Shanghai, China). UBE2S antibody (ABclonal, Wuhan, China) was 1:200 diluted.

### Establishment of ubiquitin-related lncRNA signature

The correlation between ubiquitin-related genes and lncRNAs was analyzed by the limma package of R software. With correlation coefficient > 0.3 and p< 0.001, we screened 1322 ubiquitin-related lncRNAs. Using univariate Cox regression analysis, we identified lncRNAs connected with the prognosis of Pca. Moreover, using multivariate Cox regression analysis, we identified lncRNAs to construct the PFS predictive signature. The computational formula is listed below (Coef = coefficient value; x = expression value of selected lncRNAs):


$$\text{Riskscore=}\sum_{\text{i}=1}^{\text{n}}\left(C{\text{oef}}_{i}\times \;{\text{x}}_{i}\right)$$

### Validation of ubiquitin-related lncRNA predictive signature

The patients included in this study were divided into high- and low-risk groups in terms of the median value of the risk score. Kaplan–Meier analysis was used to analyze the PFS and other clinicopathologic variables. The survival ROC package was used to calculate the receiver operating characteristic (ROC) curves and the area under the curve (AUC) for each variable. Next, Pca patients were assigned randomly to training and validation sets with a ratio of 1:1, and the same process of validation was carried out in each set. A nomogram was derived from the risk score combined with the clinicopathological factors such as age, Gleason score, T stage, and M stage to predict the PFS. The predicted PFS of the nomogram was contrasted with the actual PFS by the calibration curve. We next used principle component analysis (PCA) to visualize the distribution of patients based on the whole genes, ubiquitin-related genes, ubiquitin-related lncRNAs, and the lncRNAs in our signature. Gene set enrichment analysis (GSEA) with KEGG data was performed to analyze the functional enrichment in the high- and low-risk groups.

### Clinical application of predictive signature

To evaluate the clinical value, we contrasted the expression levels of immune functions, cell types, and checkpoints between the high- and low-risk groups. The data of immune checkpoint were derived from the ImmPort database (https://www.immport.org/). The enrichment analysis of the most significantly expressed immune checkpoints was performed with the Metascape website (http://www.metascape.org/). The half-maximal inhibitory concentration (IC50) of drugs was calculated. Wilcoxon test was adopted to compare the IC50 levels between the high- and low-risk groups.

## Results

### Enrichment analysis of ubiquitin-related genes

**Figure 1:**
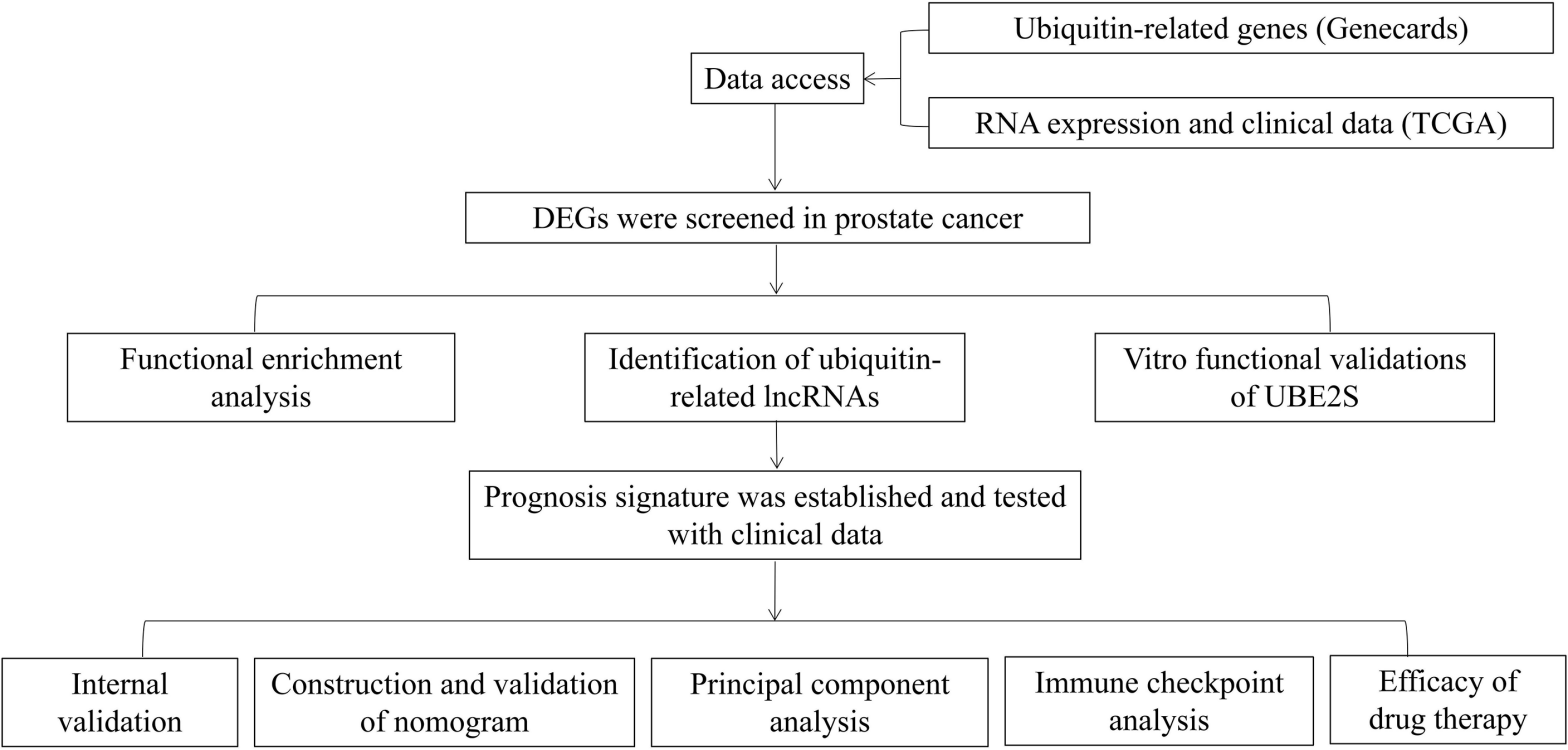
Flowchart of this research.

**Figure 2:**
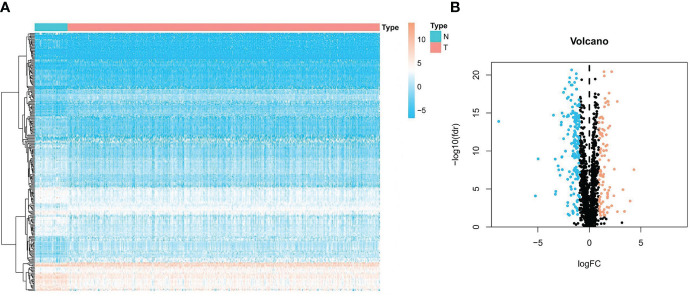
Expression of ubiquitin-related DEGs in Pca and normal prostate tissue. **(A)** Heatmap of ubiquitin-related genes in Pca. **(B)** Volcano plot of ubiquitin-related genes in Pca.

**Figure 3:**
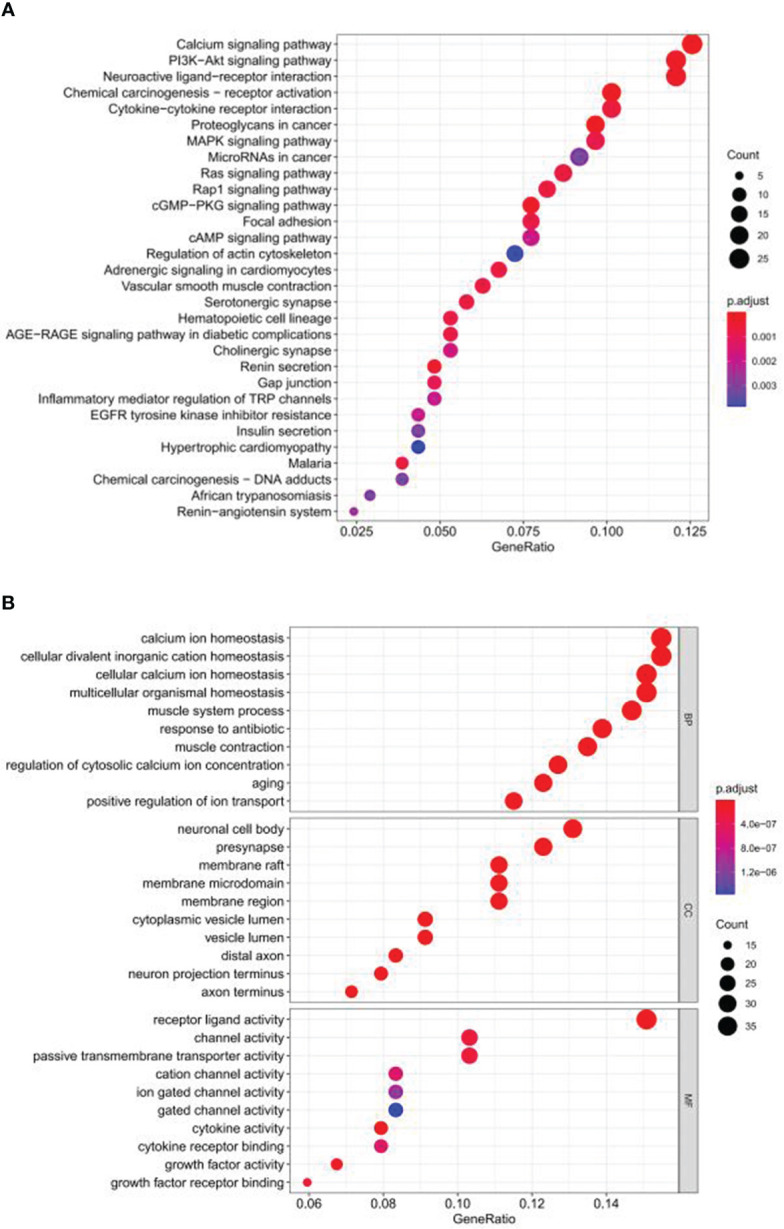
Functional enrichment analysis of ubiquitin-related DEGs in Pca based on KEGG and GO. **(A)** KEGG. **(B)** GO.

The flowchart of our research is displayed in **Figure 1**. A total of 254 genes were filtered as ubiquitin-related DEGs in Pca, namely 92 upregulated genes and 162 downregulated genes (**Figures 2A, B**). KEGG enrichment analysis indicated that ubiquitin-related DEGs were significantly enriched in the calcium signaling pathway, the PI3K-Akt signaling pathway, the neuroactive ligand–receptor interaction, etc. (**Figure 3A**). In the aspect of the biological process, GO analysis showed that DEGs were mainly enriched in calcium ion homeostasis, cellular divalent inorganic cation homeostasis, cellular calcium ion homeostasis, etc. In the aspect of cellular components, DEGs were enriched in neuronal cell body, membrane raft, membrane microdomain, etc. In the aspect of molecular function, DEGs were enriched in receptor ligand activity, channel activity, passive transmembrane transporter activity, etc. (**Figure 3B**).

### Biological validation of UBE2S

**Figure 4:**
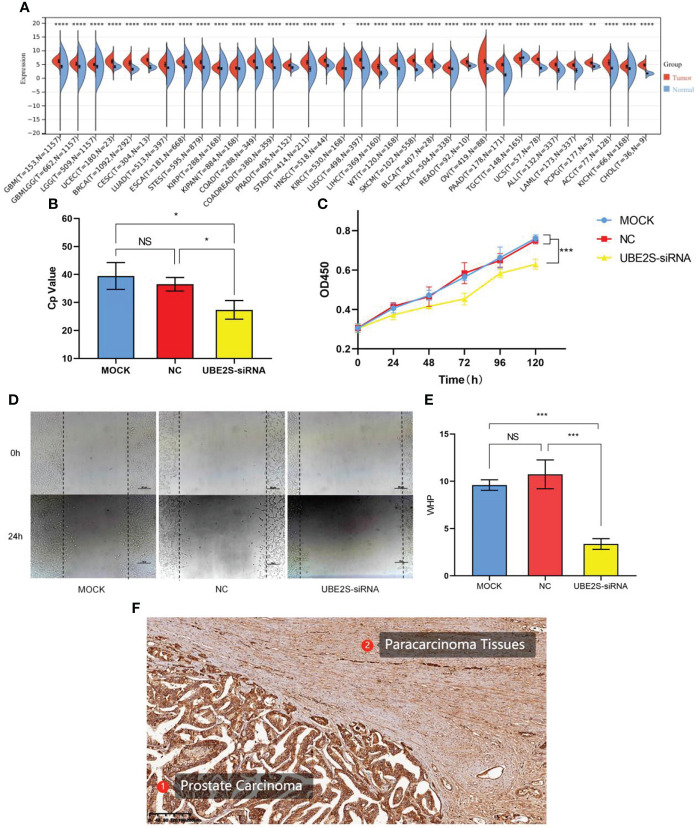
Pan-cancer analysis and functional validations of UBE2S. **(A)** Pan-cancer analysis of UBE2S. **(B)** PCR analysis of transfection efficiency. **(C)** CCK8 cell proliferation curve. UBE2S suppression inhibits proliferation of LNCaP cells (p< 0.001). **(D, E)** Scratch-wound-healing assay in LNCaP cells. Wound-healing percentage showed a significant decrease after UBE2S inhibition (p< 0.001). **(F)** Immunohistochemistry analysis of UBE2S in Pca and adjacent tissue. GBM, glioblastoma multiforme; GBMLGG, glioma; LGG, brain lower grade glioma; UCEC, uterine corpus endometrial carcinoma; BRCA, breast invasive carcinoma; CESC, cervical squamous cell carcinoma and endocervical adenocarcinoma; LUAD, lung adenocarcinoma; ESCA, esophageal carcinoma; STES, stomach and esophageal carcinoma; KIRP, kidney renal papillary cell carcinoma; KIPAN, pan-kidney cohort (KICH+KIRC+KIRP); COAD, colon adenocarcinoma; COADREAD, colon adenocarcinoma/rectum adenocarcinoma esophageal carcinoma; PRAD, prostate adenocarcinoma; STAD, stomach adenocarcinoma; HNSC, head and neck squamous cell carcinoma; KIRC, kidney renal clear cell carcinoma; LUSC, lung squamous cell carcinoma; LIHC, liver hepatocellular carcinoma; SKCM, skin cutaneous melanoma; BLCA, bladder urothelial carcinoma; THCA, thyroid carcinoma; READ, rectum adenocarcinoma; OV, ovarian serous cystadenocarcinoma; PAAD, pancreatic adenocarcinoma; TGCT, testicular germ cell tumors; UCS, uterine carcinosarcoma; LAML, acute myeloid leukemia; PCPG, pheochromocytoma and paraganglioma; ACC, adrenocortical carcinoma; KICH, kidney chromophobe; CHOL, cholangiocarcinoma; *p< 0.05; **p< 0.01; ***p< 0.001; ****p< 0.0001 ns, non-significant; MOCK, mock transfection group; NC, negative control group; UBE2S-siRNA, UBE2S suppression group.

Pan-cancer analysis of UBE2S is shown in **Figure 4A**. A significant difference in the expression of UBE2S was observed in a variety of human cancer diseases (p< 0.0001 in prostate adenocarcinoma). The result of real-time PCR illustrated that siRNA that specifically targeted UBE2S significantly inhibited the expression of UBE2S (p< 0.05) (**Figure 4B**). Our results demonstrated that the growth rate of LNCaP cells was significantly decreased in the UBE2S-siRNA group compared with the MOCK or NC group (**Figure 4C**). The results of scratch-wound-healing assay showed that suppressed UBE2S induced obviously decreased migratory ability (p< 0.001) (**Figures 4D, E**). These results suggested that UBE2S had the ability to promote proliferation and migratory in Pca cells. The paraffin-embedded sections of Pca patients were used for immunohistochemical detection of UBE2S expression. We found that the positive staining intensity of UBE2S in Pca tissues was significantly stronger than in the corresponding adjacent tissues (**Figure 4F**).

### Construction of ubiquitin-related lncRNA predictive signature

**Figure 5:**
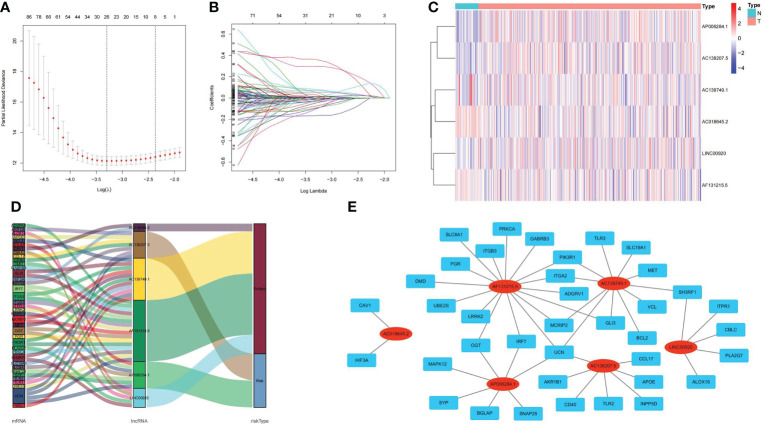
Visualizations of LASSO regression model, expression levels, and lncRNA–mRNA network of ubiquitin-related lncRNAs. **(A)** Screening the optimal partial likelihood deviance. **(B)** Coefficient estimates for Pca patients. **(C)** Distribution heatmap of ubiquitin-related lncRNAs in signature. **(D)** Sankey diagram of prognostic lncRNAs. **(E)** Co-expression network of prognostic lncRNAs.

A total of 1322 ubiquitin-related lncRNAs were filtered. From these, 215 lncRNAs were identified as independent prognosis factors for Pca by univariate Cox regression analysis. Even further, six lncRNAs (LINC00920, AC139749.1, AF131215.5, AP006284.1, AC138207.5, and AC018645.2) were screened to construct a predictive signature by LASSO regression analysis. The visualization of LASSO regression is shown in **Figures 5A, B**. The expression of six ubiquitin-related lncRNAs is shown in **Figure 5C**. The Sankey plot and the co-expression network of lncRNA-mRNA were obtained by ggalluvial R software package and Cytoscape software (Ver 3.6.1). LINC00920, AC139749.1, AF131215.5, and AC018645.2 were protective factors of Pca, while AP006284.1 and AC138207.5 were risk factors of Pca (**Figure 5D**). The strong co-expression relationships (p< 0.001) were observed in AC018645.2 and 2 genes (CAV1 and HIF3A), in AF131215.5 and 16 genes (DMD, PGR, SLC8A1, ITGB3, PRKCA, GABRB3, PIK3R1, ITGA2, ADGRV1, GLI3, MCRIP2, UCN, IRF7, OGT, LRRK2 and UBE2S), in AC139749.1 and 11 genes (MET, SH3RF1, VCL, BCL2, GLI3, UCN, MCRIP2, ITGA2, PIK3R1, TLR3, and SLC19A1), in AP006284.1 and 7 genes (SYP, BGLAP, ANAP25, UCN, IRF7, OGT, and MAPK12), in AC138207.5 and 7 genes (UCN, AKR1B1, CD40, TLR2, INPP5D, APOE, and CCL17), and in LINC00920 and 5 genes (SH3RF1, ITPR1, CBLC, PLA2G7, and ALOX15) (**Figure 5E**).

### Validation of ubiquitin-related lncRNA predictive signature

**Figure 6:**
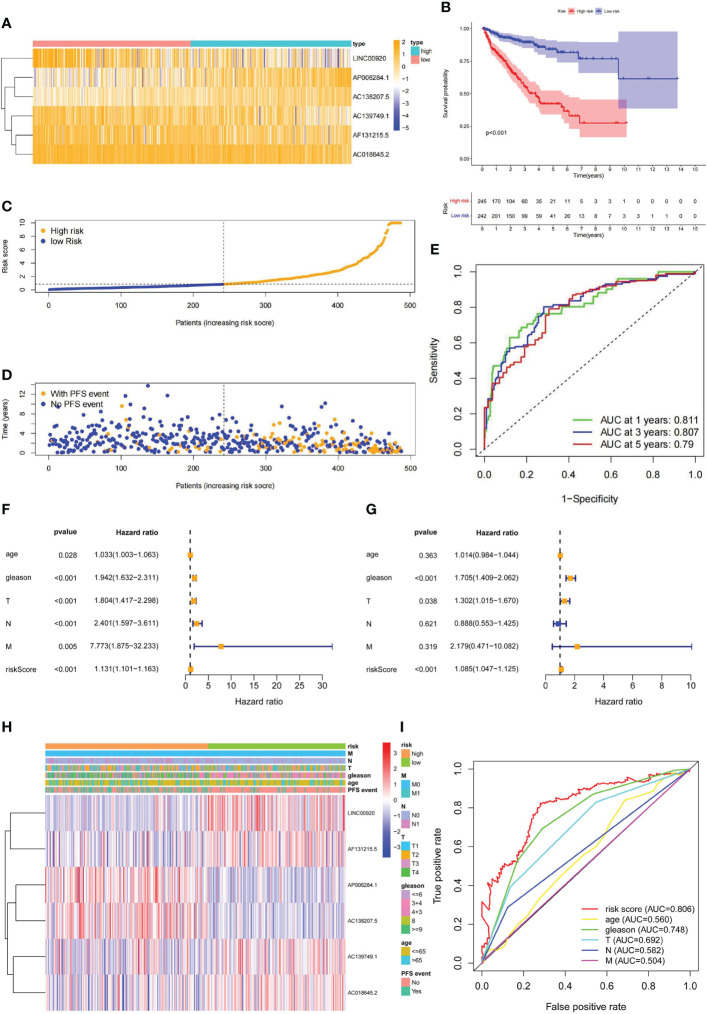
The prediction validation of ubiquitin-related lncRNAs signature. **(A)** Distribution heatmap of six prognostic ubiquitin-related lncRNAs. **(B)** Kaplan–Meier analysis of PFS between high- and low-risk groups. **(C)** Distribution of the risk score in Pca patients. **(D)** Distribution of Pca patients with or without PFS events. **(E)** ROC curve and AUCs at 1-, 3-, and 5-year PFS (AUC at 1 year = 0.811, at 3 years = 0.807, at 5 years = 0.790). **(F)** Forest plot of univariate Cox regression analysis. **(G)** Forest plot of multivariate Cox regression analysis. **(H)** Heatmap of ubiquitin-related lncRNAs and clinicopathological variables. **(I)** ROC curve of the risk score and clinicopathological variables.

According to the median value of the risk score calculated from our lncRNA predictive signature, the Pca patients were grouped into two subgroups (high and low risk). The heatmaps showed distinct lncRNA expression profile. In the low-risk group, expressions of LINC00920, AC139749.1, AF131215.5, and AC018645.2 were high. In the high-risk group, expressions of AP006284.1 and AC138207.5 were high (**Figure 6A**). Kaplan–Meier analysis showed that the PFS in the high-risk group was significantly shorter than that in the low-risk group (p< 0.001) (**Figure 6B**). The distribution of the risk score among Pca patients is shown in **Figure 6C**. The Pca patients with high risk scores seem to be more likely to experience PFS events (**Figure 6D**). The ROC curves demonstrated excellent AUC values for 1-, 3-, and 5-year PFS (0.811, 0.807, and 0.790, respectively) (**Figure 6E**). Univariate Cox regression analysis revealed age (p< 0.05), Gleason score (p< 0.001), T stage (p< 0.001), N stage (p< 0.001), M stage (p< 0.05), and risk score (p< 0.001) as the independent predictors of PFS in Pca patients (**Figure 6F**). We followed this analysis with a multivariate Cox regression analysis, which showed Gleason score (p< 0.001), T stage (p< 0.05), and risk score (p< 0.001) as the independent predictors (**Figure 6G**). Clinicopathological information of the Pca patients grouped by lncRNAs is exhibited in the heatmap (**Figure 6H**). In **Figure 6I**, the risk score displayed a significantly higher AUC value (AUC = 0.806) compared to age (AUC = 0.560), Gleason score (AUC = 0.748), T stage (AUC = 0.692), N stage (AUC = 0.582), and M stage (AUC = 0.504).

### Prediction ability in subgroups of clinicopathological variables

**Figure 7:**
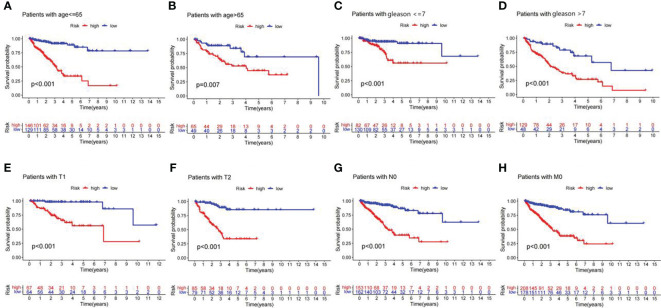
Kaplan–Meier curves of PFS in Pca patients grouped by clinicopathological categories. **(A)** Age<= 65 years. **(B)** Age > 65 years. **(C)** Gleason<= 7. **(D)** Gleason > 7. **(E)** T1 stage. **(F)** T2 stage. **(G)** N0 stage. **(H)** M0 stage.

Stratification survival analysis was used to evaluate the prediction ability of the lncRNA signature in different clinicopathological subgroups. In subgroups of age<= 65, age > 65, Gleason score<= 7, Gleason score > 7, T1 stage, T2 stage, N0 stage, and M0 stage, the PFS of patients in the high-risk group was significantly worse than that in the low-risk group (**Figures 7A–H**). The results suggest that the lncRNA predictive signature is applicable for the prognosis prediction of Pca patients without the aid of clinicopathological variables.

### Internal validation of ubiquitin-related lncRNA signature

**Figure 8:**
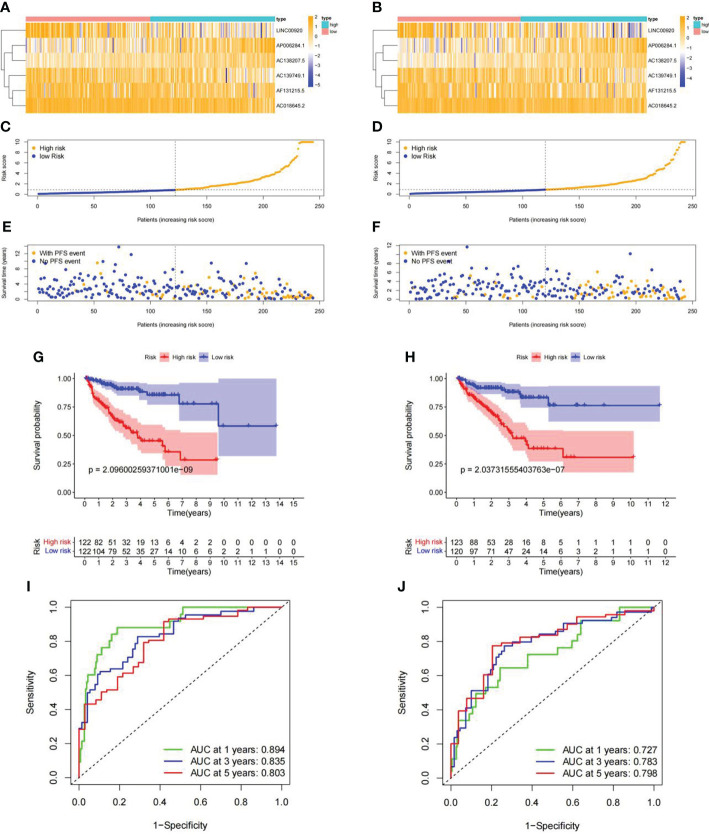
Internal validation of ubiquitin-related lncRNA prognosis signature. **(A, B)** Distribution heatmap of ubiquitin-related lncRNAs in the training and validation sets. **(C, D)** Distribution of the risk score in the training and validation sets. **(E, F)** Distribution of PFS events in the training and validation sets. **(G, H)** Kaplan–Meier survival curve in the training and validation sets. **(I, J)** ROC curve and AUCs of PFS at 1, 3, and 5 years in the training and validation sets.

To further verify the predictive value of this signature, the Pca patients were randomly divided into the training set (n = 244) and the validation set (n = 243) in a ratio of 1:1. The heatmaps displayed the demographic characteristics of patients in the training set (**Figure 8A**) and the validation set (**Figure 8B**). The distribution of risk score and PFS events among patients in the training set is shown in **Figures 8C, E**. In the validation set, the result of the distribution is shown in **Figures 8D, F**. A higher risk score was accompanied by more PFS events in both sets. Kaplan–Meier curves demonstrated a significantly lower PFS probability (p = 2.096e-09) of patients with high risk scores in the training set (**Figure 8G**). Similar result (p = 2.037e-07) was obtained in the validation set (**Figure 8H**). The ROC curves of each set showed favorable predictive performance. In the training set, the AUCs of 1-, 3-, and 5-year PFS were 0.894, 0.835, and 0.803, respectively (**Figure 8I**). In the validation set, the AUCs of 1-, 3-, and 5-year PFS were 0.727, 0.783, and 0.798, respectively (**Figure 8J**).

### Construction and validation of nomogram

**Figure 9:**
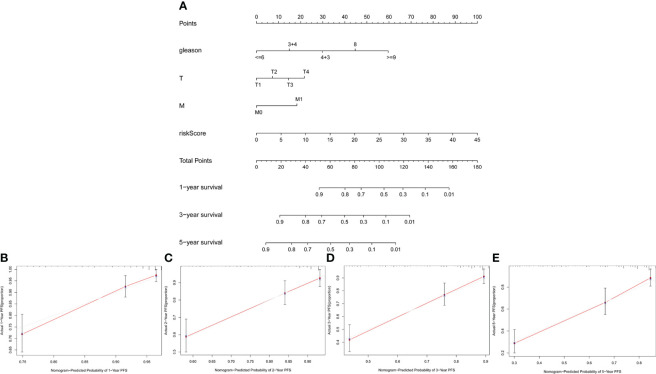
Construction and validation of nomogram. **(A)** Nomogram based on clinicopathological variables and risk score. **(B–E)** Calibration curves for predicting PFS at 1, 2, 3, and 5 years.

To verify the clinical value of our risk scoring system, we built a nomogram to predict the 1-, 3-, and 5-year PFS of Pca patients (**Figure 9A**). The calibration curves of 1-, 2-, 3-, and 5-year PFS demonstrated favorable consistency between the actual and predicted data (**Figures 9B–E**).

### Principal components and pathway enrichment analysis

**Figure 10:**
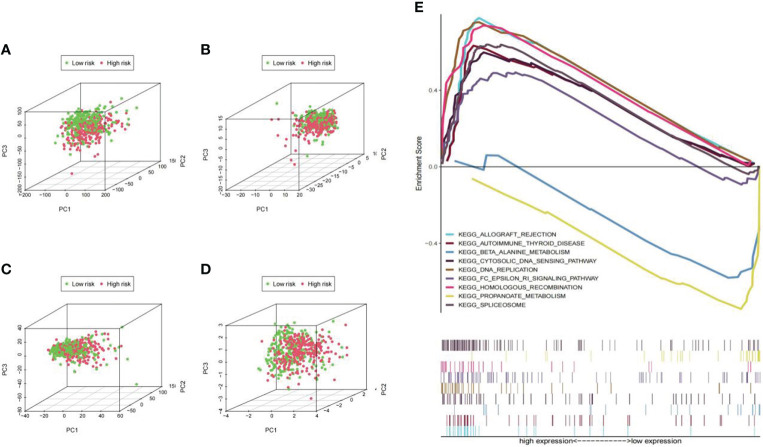
PCA maps for Pca patients and pathway enrichment analysis in low- and high-risk groups. **(A)** PCA according to all genes; **(B)** PCA according to ubiquitin-related genes; **(C)** PCA according to ubiquitin-related lncRNAs; **(D)** PCA according to lncRNAs in our signature. The separation of the red and green dots becomes stronger in this set. **(E)** Significant enrichment was identified in two pathways (bate alanine metabolism and propanoate metabolism; p<0.01) in the low-risk group and seven pathways (allograft rejection, autoimmune thyroid disease, etc.; p<0.01) in the high-risk group.

The distribution of Pca patients was visualized in PCA maps according to whole genes (**Figure 10A**), ubiquitin-related genes (**Figure 10B**), ubiquitin-related lncRNAs (**Figure 10C**), and the lncRNAs included in our signature (**Figure 10D**). The red dots represent patients in the high-risk group, while the green dots represent patients in the low-risk group. The results showed that separation of the dots with red and green colors becomes stronger when taking only signature lncRNAs. In the results of GSEA, significant enrichment (p< 0.01) was identified in two pathways (bate alanine metabolism and propanoate metabolism) in the low-risk group and seven pathways (allograft rejection, autoimmune thyroid disease, cytosolic deoxyribonucleic acid (DNA) sensing pathway, DNA replication, FC epsilon RI signaling pathway, homologous recombination, and spliceosome) in the high-risk group (**Figure 10E**).

### Correlation analysis between signature and immune function

**Figure 11:**
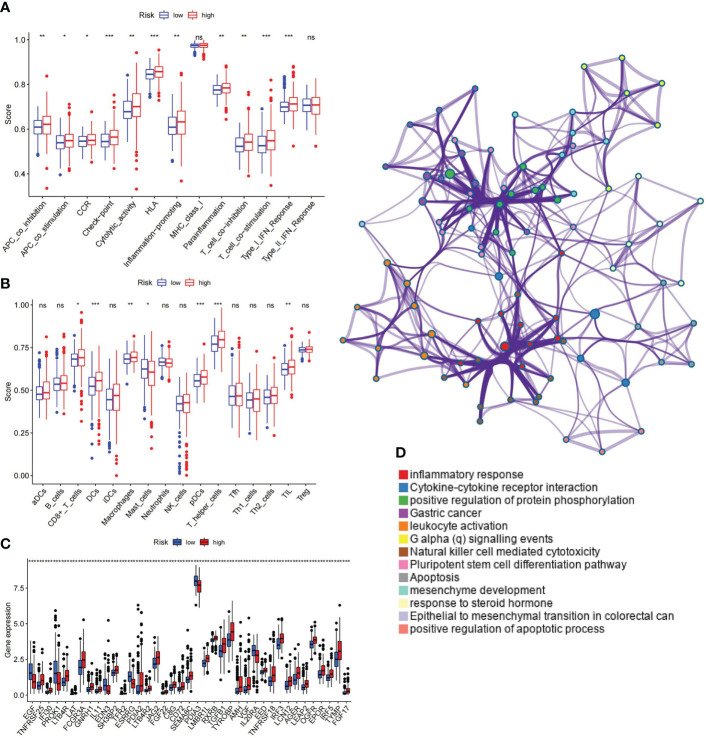
Correlation between ubiquitin-related signature and immunological parameter. **(A)** Differences of immune-related functions in high- and low-risk groups. **(B)** Infiltration levels of immune cells in high- and low-risk groups. **(C)** Immune checkpoints with maximal expression differences in high- and low-risk groups (p< 0.0001). **(D)** Functional enrichment network of immune checkpoints. APC, antigen-presenting cell; CCR, chemokine receptor; HLA, human leukocyte antigen; MHC, major histocompatibility complex; IFN, interferon; aDCs, activated dendritic cells; iDCs, immature dendritic cells; NK, natural killer; pDCs, plasmacytoid dendritic cells; Tfh, T follicular helper; Th1, T helper type 1; Th2, T helper type 2; TIL, tumor-infiltrating lymphocyte; Treg, T regulatory cell. *p< 0.05; **p< 0.01; ***p< 0.001; ns, non-significant.

Immune functions, cells, and checkpoint members were enrolled in the analysis and assessed as therapeutic targets. The enrichment results of GSEA for immune-related functional pathways were quantified. The results are presented in **Figures 11A, B**, which shows a significantly higher activation level of antigen-presenting cell (APC) co-inhibition, APC co-stimulation, chemokine receptor (CCR), cytolytic activity, human leukocyte antigen (HLA), inflammation promoting, parainflammation, T-cell co-inhibition, T-cell co-stimulation, and type-1 interferon (IFN) response in the high-risk group than in the low-risk group (**Figure 11A**). The level of CD8+ T cells, dendritic cells (DCs), macrophages, plasmacytoid dendritic cells (pDCs), T helper cells, and tumor-infiltrating lymphocyte (TIL) was distinctly higher in the high-risk group. On the contrary, mast cells exhibited a lower level in the high-risk group (**Figure 11B**). Significant differences were observed in 39 immune checkpoints (p< 0.0001) (**Figure 11C**), which were enriched in inflammatory response, cytokine–cytokine receptor interaction, positive regulation of protein phosphorylation, etc. (**Figure 11D**).

### Correlation analysis between signature and medication

**Figure 12:**
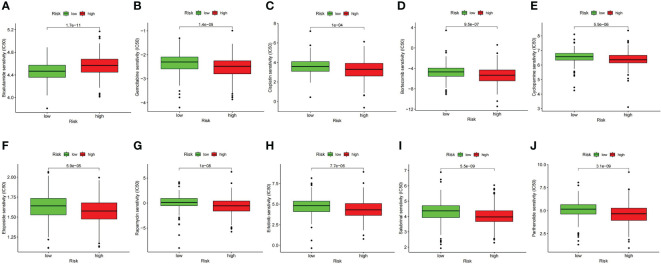
Comparison of drug sensitivity between high- and low-risk groups. **(A)** IC50 of bicalutamide. **(B)** IC50 of gemcitabine. **(C)** IC50 of cisplatin. **(D)** IC50 of bortezomib. **(E)** IC50 of cyclopamine. **(F)** IC50 of etoposide. **(G)** IC50 of rapamycin. **(H)** IC50 of erlotinib. **(I)** IC50 of salubrinal. **(J)** IC50 of parthenolide.

The contrast analysis was performed on the sensitivity of oncology drugs according to risk stratification. The IC50 of bicalutamide was lower in the low-risk group, and the IC50 of gemcitabine and cisplatin was lower in the high-risk group (**Figures 12A–C**), suggesting that our signature will contribute to exploring individualized treatment for Pca patients. To explore the new therapeutic targets, we observed that the IC50 levels of bortezomib, cyclopamine, etoposide, rapamycin, erlotinib, salubrinal, and parthenolide were distinctly lower in the high-risk group (**Figures 12D–J**). Among them, bortezomib, which is involved in ubiquitylation-mediated proteolysis, indicated meaningful therapeutic potential for Pca.

## Discussion

Because PCa is the second highest cancer in men worldwide, the prediction of prognosis has always been the focus of research. Some studies concerning prognostic markers and prediction methods of Pca have previously been reported. Song et al. noticed that ubiquitin-related genes had a high predictive ability for biochemical recurrence of PCa (22). Shao et al. combined their microarray data with the TCGA database and identified a signature with six genes to predict the progression of Pca. In this study, the AUC value used to predict early biochemical recurrence in TCGA sets was 0.73 (23). Catalona et al. presented the validity of the PHI score; one index can be calculated from pro prostate-specific antigen (PSA), free PSA, and total PSA to identify Pca with an AUC of 0.703 (24). With a similar approach, Loeb et al. obtained an AUC value of 0.707 in Pca patients with total PSA of 4–10n g/ml (25). Prostate cancer antigen 3 (PCA3), a prostate-specific non-coding RNA, as a molecular detector for Pca has been approved by the Unite States Food and Drug Administration (FDA) (26). Pepe et al. used meta-analysis and drew the conclusion that the AUC value of PCA3 ranged from 0.72 to 0.85 with a different cutoff (27). Another signature named the TMPRSS2:ERG score is based on the expression of a hybrid gene specific for Pca. Tomlins et al. combined PCA3 and the TMPRSS2:ERG score to predict Pca, and the AUC value of this prediction model was 0.693–0.729 (28). Van Neste et al. measured DNA methylation in prostate tissues and found that this method was a significant predictor of Pca with AUC=0.762 (29). Additionally, McDunn et al. calculated the prostarix risk score (PRS), which was derived from the metabolites of Pca to predict the recurrence rate of Pca with AUC from 0.53 to 0.64 (30). Inspired by the functions of ubiquitin associated with human cancer pathogenesis and the important role of lncRNAs in the regulation of gene expression, we yielded a prognosis predicting the signature of Pca based on ubiquitin-related lncRNAs for the first time. We obtained the AUC of 1-, 3-, and 5-year PFS from 0.803 to 0.894 in the training set and from 0.727 to 0.798 in the validation set. This result demonstrates that our signature has higher prediction accuracy and is able to facilitate clinical prognostic evaluation and personalized treatment.

Different from purely bioinformatic approaches, our study included *in vitro* test of the ubiquitin gene. UBE2S was significantly expressed higher in Pca tissues, and the correlation between UBE2S and Pca has not yet been defined. Therefore, we chose UBE2S as the representative gene of the ubiquitin family. Following the inhibition of UBE2S, LNCaP cell proliferation and motility were suppressed. This phenomenon suggests that UBE2S acts as an oncogene in Pca, fits to the result of pan-cancer analysis, and suggests that the ubiquitin family is involved in the pathogenesis of Pca.

GSEA analysis showed that allograft rejection, autoimmune thyroid disease, cytosolic DNA sensing pathway, DNA replication, FC epsilon RI signaling pathway, homologous recombination, and spliceosome were mainly enriched in the high-risk group. Of these, allograft rejection, autoimmune thyroid disease, and the FC epsilon RI signaling pathway are closely related to the immune function of the organism. Existing studies do not provide specific evidence for the roles of the FC epsilon RI signaling pathway in the pathogenesis of Pca. However, the FC epsilon RI signaling pathway has been shown to participate in the regulation of multiple downstream signaling pathways such as the PI3K-AKT (31) and MAPK pathways (32), which have been shown to be strongly associated with the pathogenesis of Pca (33–35). As is well known, the FC epsilon RI signaling pathway is activated by immunoglobulin E (IgE) (36). When looking for the correlation between the clinical characteristics of Pca and the IgE level, some researchers found out that IgE was inversely correlated with the risk of a higher PSA level (37). Therefore, we hypothesize that the FC epsilon RI signaling pathway, which is defined as a classical immune pathway, plays a key role in Pca pathogenesis through the activation of downstream pathways and will validate this hypothesis in future work.

In terms of human immunity, our result showed a higher activation level of antigen presentation, inflammation, and T-cell function in the high-risk group. Immune cells involved in these function such as DCs, pDCs, TIL, CD8+ T cells, and T helper cells were expressed at higher levels in the high-risk group simultaneously. Similar conclusions have been obtained by other researchers: the infiltration of CD4+ T cells, CD8+ T cells, and DCs was positively correlated with colon adenocarcinoma and stomach adenocarcinoma (38); the intratumoral-activated cDCs were increased by the fascin inhibitor NP-G2-044 (39); and a high CD8+ T-cell level was correlated with the low expression of YKT6 in oral squamous cell carcinoma (40). These studies have also supported the validity of our prognosis signature. In addition, we found 39 immune checkpoints with the largest expression difference between the low- and high-risk groups. Enrichment analysis revealed that these 39 genes were mostly enriched in inflammatory response, cytokine–cytokine receptor interaction, and positive regulation of protein phosphorylation. It can provide clues to seek the relationship between immune activity and Pca.

As for the therapy, we found that high-risk Pca patients are probably sensitive to conventional chemotherapy drugs (gemcitabine and cisplatin) and drugs applied mainly to the treatment of cancer in other organs (bortezomib, cyclopamine, etoposide, rapamycin, erlotinib, salubrinal, and parthenolide). This finding suggested that our signature might be effective for searching novel therapeutic targets for Pca. Particularly, bortezomib acted directly on the ubiquitin–proteasome system and has been approved by the FDA in treating multiple myeloma and mantle cell lymphoma (41). With the ubiquitin-related pharmacological function, bortezomib exhibits great potential in Pca treatment. Another interesting result is the drug resistance of bicalutamide in high-risk patients. Once Pca patients who were treated with androgen deprivation therapy progressed to the CRPC stage, bicalutamide was gradually ineffective (42). Our signature was proved to be consistent with the actual phenomenon in clinical practice.

However, there are still some deficiencies in our study. The cohorts from other databases were excluded due to the limitation of available clinical information. We can only use the data from the TCGA database for internal validation. To address this question, we plan to improve our own cohort data by RNA sequencing and perform external validation. Additionally, the mechanism of the ubiquitin-related lncRNAs in Pca remains unclear. We will verify the mechanisms by experiments in the future.

In conclusion, our ubiquitin-related lncRNA signature can efficiently predict the prognosis and bring novel therapeutic targets for Pca patients.

## Data availability statement

The datasets presented in this study can be found in online repositories. The names of the repository/repositories and accession number(s) can be found in the article/supplementary material.

## Author contributions

XL, JW, and LY contributed to the conception of the study. XL, LJ, XS, and ZZ performed the experiment. WM, ZZ, XS, LH and GY contributed significantly to analysis and manuscript preparation. XL performed the data analyses and wrote the manuscript. WM, LJ, and ZZ helped perform the analysis with constructive discussions. All authors contributed to the article and approved the submitted version.

## Funding

This work was supported by grants from the Health System Independent Innovation Science Foundation of Shanghai Putuo District (No.ptkwws201819), the National Natural Science Foundation of China (No.81672549 and No.81972409), and the Natural Science Foundation of Shanghai (No.15ZR1433000).

## Conflict of interest

The authors declare that the research was conducted in the absence of any commercial or financial relationships that could be construed as a potential conflict of interest.

## Publisher’s note

All claims expressed in this article are solely those of the authors and do not necessarily represent those of their affiliated organizations, or those of the publisher, the editors and the reviewers. Any product that may be evaluated in this article, or claim that may be made by its manufacturer, is not guaranteed or endorsed by the publisher.

## Abbreviation

lncRNA, long noncoding ribonucleic acid; Pca, prostate cancer; TCGA, The Cancer Genome Atlas; DEGs, differentially expressed genes; PFS, progression-free survival; ROC, receiver operating characteristic; AUC, area under the curve; CRPC, castration-resistant prostate cancer; mRNA, messenger RNA; miRNA, micro RNA; KEGG, Kyoto Encyclopedia of Genes and Genomes; GO, Gene Ontology; PCR, polymerase chain reaction; PCA, principle components analysis; GSEA, gene set enrichment analysis; IC50, half-maximal inhibitory concentration; DNA, deoxyribonucleic acid; APC, antigen-presenting cell; CCR, chemokine receptor; HLA, human leukocyte antigen; MHC, major histocompatibility complex; IFN, interferon; DCs, dendritic cells; pDCs, plasmacytoid dendritic cells; TIL, tumor-infiltrating lymphocyte; PSA, prostate-specific antigen; PCA3, prostate cancer antigen 3; FDA, Food and Drug Administration; PRS, prostarix risk score; IgE, immunoglobulin E.
